# Plasma lipid profiling and diagnostic biomarkers for oral squamous cell carcinoma

**DOI:** 10.18632/oncotarget.21289

**Published:** 2017-09-27

**Authors:** Lina Wang, Xin Wang, Ying Li, Yan Hou, Fengyu Sun, Shuang Zhou, Chunming Li, Bin Zhang

**Affiliations:** ^1^ Department of Oral and Maxillofacial Surgery, The Second Affiliated Hospital of Harbin Medical University, Harbin 150001, Heilongjiang, China; ^2^ Institute of Hard Tissue Development and Regeneration, The Second Affiliated Hospital of Harbin Medical University, Harbin 150001, Heilongjiang, China; ^3^ Heilongjiang Academy of Medical Sciences, Harbin 150001, Heilongjiang, China; ^4^ Department of Plastic and Maxillofacial Surgery, Heilongjiang Province Hospital, Harbin 150001, Heilongjiang, China; ^5^ Department of Statistics Sciences, Harbin Medical University, Harbin 150001, Heilongjiang, China

**Keywords:** oral squamous cell carcinoma, lipidomics, plasma, diagnosis, early diagnosis

## Abstract

Biological requirements for tumor cell proliferation include the sustained increase of structural, energetic, signal transduction and biosynthetic precursors. Because lipids participate in membrane construction, energy storage, and cell signaling. We hypothesized that the differences in lipids between malignant carcinoma and normal controls could be reflected in the bio-fluids. A total of 100 pre-operative plasma samples were collected from 50 oral squamous cell carcinoma (OSCC), 50 normal patients and characterize by lipid profiling using ultra performance liquid chromatography/electro spray ionization mass spectrometry (UPLC-MS). The lipid profiles of the OSCC and control samples as well as the different stages were compared. Differentially expressed lipids were categorized as glycerophospholipids and sphingolipids. All glycerophospholipids were decreased, especially phosphatidylcholine and phosphoethanolamine plasmalogens, whereas sphingolipids were increased in the OSCC patients compared to the controls. We further identified 12 staging related lipids, which could be utilized to discriminate early stage patients from advanced stage patients. In the future, the differential lipids may provide biologists with additional information regarding lipid metabolism and guide clinicians in making individualized therapeutic decisions if these results are confirmed in a larger study.

## INTRODUCTION

Although enormous progress has been made in cancer research during the past decades, oral squamous cell carcinoma (OSCC) remains a malignancy worldwide. Due to the lack of public awareness and screening method, patients are often diagnosis at an advanced stage accompanied by metastasis, which leads to a poor prognosis. The five-year survival rate of this disease is below 50% [1, 2], which is strongly dependent upon the stage at OSCC diagnosis. When OSCC patients are diagnosis at early stage of development, it has less disfiguring and psychologically-traumatic and with a 5-year survival rate approaching to 90% [3]. The outcome of this disease is significantly better if patients could be diagnosed at the early stage and received the individual treatment plan based on biological malignancy. However, currently histopathology grade, lymph node (LN) metastasis, and serum tumor markers, such as serum squamous cell carcinoma antigen (SCCA), are still used as prognostic factors for patients with OSCC. Meanwhile, although the higher level of serum SCCA may serve as a marker for dysplasia and progression to OSCC, the predictive performance for abnormal level of SCCA for OSCC was not satisfactory (*P*=0.055) [2]. In recent years, considerable progress has been made in understanding the genetic and protean basis of OSCC and identified a number of potential biomarkers that might improve the diagnostic and predictive performance [4–6]. However, no ideal biomarkers have been widely used in clinical practice because of poor validation performance and expenses. Because metabolites are the ultimate downstream product of gene expression, changes in metabolites are amplified relative to the changes in the transcriptome and proteome [7] and might be the most similar to the phenotype of the biological system studied. To date, some metabolites have been reported as biomarkers in the diagnosis of OSCC [8, 9]. However, these studies focused on conducting metabolomics on tissues and cell line, not plasma for diagnosis. Meanwhile, in such an extremely complex pathological process, it requires adaptations across multiple metabolic process to satisfy the energy in order to increase the proliferation rate. Therefore, developing new or supplementary metabolites for early diagnosis is urgently needed.

In all living cells, lipids are needed to maintain cellular structure, store energy and involved in cell signaling. Lipid metabolism connects to signaling networks in the regulation of cell growth, proliferation, differentiation apoptosis and membrane homeostasis [10–12]. Additionally, deregulated lipid metabolism can alter membrane composition and permeability, which might cause the development and progression of many diseases, especially malignant carcinoma. Therefore, theoretically, lipid profiles in cancer cells could be distinguishable from those of normal cells, and such a distinctive lipid profile could be reflected in the biofluids of patients with OSCC. Lipidomics, a specific component of metabolomics [13], describes the identification, quantification and profiling of individual lipid molecules extracted from biological samples [14]. It has been widely utilized to diagnose and investigate the pathogenesis of various cancers, such as pancreatic adenocarcinoma [15], thyroid cancer [16], colon cancer [17], hepatocellular carcinoma [18], glioblastoma [19] and prostate cancer [20]. However, few studies have been performed to systematically investigate plasma lipid profiling and to comprehensively characterize the changes in lipid metabolism between OSCC patients and controls.

In the present study, we aimed to expand the metabolic process to lipidomics study on plasma samples of OSCC and compared the lipid profiles of OSCC patients and controls. Furthermore, differentially expressed lipids were identified between OSCC patients and controls. Finally, the lipids associated with specific pathological stages were presented in order to select the biomarkers for OSCC early diagnosis.

## RESULTS

### Demographics and clinic pathological characteristics

We performed case-control matching for patients and controls. A total of 100 patients eligible for analysis, 50% (50) with malignant tumors, 50% (50) control, were enrolled in this study from the Department of Oral and Maxillofacial Surgery, The affiliated Second Hospital of Harbin Medical University, Harbin, Heilongjiang Province, China. All patients had pathological confirmation for diagnosis, metastasis assessment and staging (I–IV). The demographics and clinic pathological characteristics of these patients were listed in Table 1. A total of 38% (19/50) of the patients were classified as stage I, 26.00% (13/50) as stage II, 26.00% (13/50) as stage III and 10.00% (5/50) as stage IV.

**Table 1:** Demographics and clinic pathological characteristics between OSCC and control groups

Characteristics	OSCC	Control
Age	45±4.22	47±4.56
Gender		
Male	43(0.86)	40(0.80)
Female	7(0.14)	10(0.20)
FIGO		
I	10(0.20)	
II	18(0.36)	
III	13(0.26)	
IV	9(0.18)	
Lymphatic metastasis		
Yes	17(0.34)	
No	33(0.66)	
Tumor location		
Carcinoma of the tongue	18(0.36)	
The floor of mouth	16(0.32)	
The buccal mucosa	5(0.10)	
The gingiva	3(0.06)	
The lip	8(0.16)	

### Plasma lipid profiling of OSCC patients and controls

**Figure 1:**
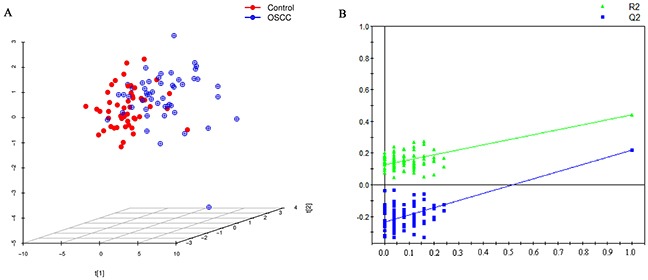
**(A)** PLS-DA score plot for discriminating OSCC and control with R^2^Y=0.443, Q^2^=0.221. **(B)** Validation plot for discriminating OSCC and control, all the permutation cumulative Q^2^ values were lower than the original values.

After excluding isotopic peaks, plasma lipid profiling consisted of 1937 ions (peaks) in positive electrospray ionization (ESI+) mode. According to the identification strategy described in previous studies [21, 22], 459 plasma lipids were used for further analysis. The principal component analysis (PCA) performed on all the samples revealed that the quality control (QC) samples were tightly clustered, which indicated the robustness of our lipid profiling platform (Supplementary Figure 1). Projection to latent structures discriminant analysis (PLS-DA) score-plot was used for classification of the plasma samples based on these differential plasma lipids (Figure 1). This analysis indicated that OSCC patients and controls had different lipid profiles. The cumulative R^2^Y and Q^2^ were 0.443 and 0.221, respectively, when three components were calculated. Validation plot obtained from 100 permutation tests showed no over-fitting of established model and all the permutation cumulative Q^2^ values were lower than the original values.

### Differential lipid selection in OSCC patients and controls

Following successful establishment of PLS-DA model, variable importance in the projection (VIP) value for each plasma lipid was calculated. Based on the two criteria of ßßlocal false discovery rate (lfdr) <0.05 and VIP>1, a total of 20 differential lipids were selected as potential lipid biomarkers of OSCC for further analysis (Supplementary Figure 2). Details about these lipids were listed in Table 2. The identified 20 plasma lipids were grouped into two lipid categories, i.e., glycerphospholipids [GPs; glycerophosphocholines (PCs), glycerophosphoethanolamines (PEs), glycerophosphoglycerols (PGs), lyso-glycerophosphocholines (Lyso-PCs)], sphingolipids [SPs; ceramides (Cers) and sphingomyelins (SMs)]. We provided several representative figures of mass spectra for each lipid categories in OSCC patients (Supplementary Figures 3–7). Interestingly, we found that all the PCs contained two acyl chains and all the PEs were with plasmalogen (pPEs). All the GPs were decreased in the OSCC patients compared with the controls. Whereas, sphingolipids were significantly increased in the OSCC patients compared with the controls.

**Table 2:** Twenty differential lipids between OSCC and control

Name	mz	rt	FC	density	*P* value	AUC
PC(32:2)	730.5203	828.82	0.72	down	<0.001	0.79
PC(34:4)	754.5129	813.36	0.56	down	<0.001	0.79
PC(36:4)	782.5313	848.59	0.69	down	<0.001	0.74
PC(36:5)	780.571	801.89	0.61	down	<0.001	0.71
PC(36:7)	776.4328	812.12	0.51	down	<0.001	0.77
PC(38:6)	806.6107	840.11	0.75	down	<0.001	0.74
PC(38:8)	802.4852	798.25	0.57	down	<0.001	0.77
PC(38:9)	800.5383	797.62	0.64	down	<0.001	0.74
PC(40:8)	830.645	829.96	0.78	down	<0.001	0.72
PE(P-34:2)	700.6135	936.65	0.76	down	<0.001	0.73
PE(P-36:2)	728.504	991.14	0.81	down	<0.001	0.7
PE(P-36:4)	724.5517	929.04	0.75	down	<0.001	0.73
PE(P-38:5)	750.4511	934.49	0.81	down	<0.001	0.66
Cer(d18:1/16:0)	538.5502	954.4	1.56	up	<0.001	0.76
Cer(d18:1/18:0)	566.5151	1007.19	1.61	up	<0.001	0.71
GlcCeramide(d18:1/16:0)	700.5802	886.65	1.61	up	<0.001	0.77
SM(d18:0/18:1)	731.6491	925.12	1.47	up	<0.001	0.79
Trihexosylceramide (d18:1/16:0)	1024.7369	830.21	1.45	up	<0.001	0.85
LysoPC(14:0)	468.3542	111.67	0.63	down	<0.001	0.77
PG(34:2)	747.5116	928.66	0.78	down	<0.001	0.71

Measured mass to charge ratio (m/z); Retention time (s, RT); Fold change (FC).

### Significance of lipids in the pathological staging of OSCC

**Figure 2:**
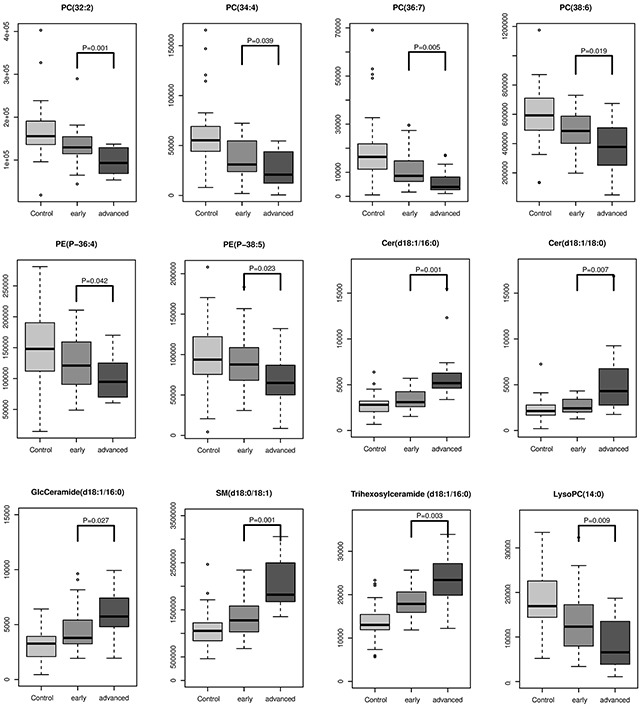
The intensity levels of lipids associated with pathological staging

Accurate staging greatly facilitates medical management and improves clinical outcomes. To further explore whether some of these differential lipids species were associated with pathological staging, we investigated the 20 differential metabolites that discriminate between early stages (I and II) and advanced stages (III and IV) and found 12 metabolites were staging-related. All GPs including PC, PE and LPC were negatively associated with pathological stage, while SMs and Cers were positively associated with pathological stages. Changing patterns of differential lipids from control across early stage and advanced stage (Figure 2).

## DISCUSSION

Lipidomics, a specific component of metabolomics, participates in constructing cell membranes, storing energy, cell differentiation and cell signaling. In the current study, a UPLC/MS plasma lipidomics method was used to systematically investigate dysregulated lipid metabolism between OSCC patients and matched normal controls. Our results suggest that the plasma lipid profiles analyzed by UPLC-QTOF/MS could be used to discriminate OSCC from controls. Twenty potential lipids are grouped into PCs, PEs, PG, Lyso-PC, Cer and SMs and related with OSCC. In addition, twelve lipids associated with pathological staging could be used for the early diagnosis of OSCC. These results suggested that these differentially expressed lipid species could reflect the development and progression of OSCC. However, dynamic alterations of differentially expressed lipid species and large-scale cohort studies should be needed to provide more accurate information for OSCC mechanism and early diagnosis in clinical practice.

All identified glycerphospholipids consisting of Lyso PCs, PCs, PEs and PGs in current study were decreased in OSCC patients compared to controls. We found that LysoPC(14:0) had a step-wise decrease with the development and progression of OSCC. As we know, LysoPC is an important signaling molecule in regulating cellular proliferation, inflammation and cancer cell invasion [23] and it is catalyzed by lysophospholipase D and generated lysophosphatidic acid (LPA), which is an important extracellular signaling molecule that controls various cell activities such as cell division and cell movement [24]. Lysophospholipase D has been reported to increase in several cancers, such as breast, prostate and ovarian cancers [25–27]. These results might partly explain the possible mechanism for the decreased plasma LysoPC levels in OSCC patients.

PC, as the main plasma membrane phospholipids, accounts for approximately 50% of total cellular phospholipids and is the most abundant phospholipids in mammalian membranes [28]. In present study, nine polyunsaturated PCs were differentially expressed between OSCC patients and controls and four of them had a step-wise decrease with the development and progression of OSCC, namely PC(32:2), PC(34:4), PC(36:7) and PC(36:6). A previous study showed that significant differences in serum levels of PC and fatty acids between advanced prostate cancer cases and controls [29]. Yin et al. utilized PC combined with LysoPC to predict the occurrence of cervical cancer and got an AUC value of 0.97 [30]. Uchiyama et al. identified PC(16:0/16:1) and PC(18:1/20:4) could discriminate the border between the cancer and stromal regions of OSCC [31]. Studies indicated that the phosphatidylethanolamine *N*-methyltransferase (PEMT) pathway is one way to synthesize PC [32], which occurs primarily in the liver via the conversion of PE to PC to satisfy the normal cellular requirement for membrane synthesis [33]. PEMT activity was decreased in cancers [18, 34], which might lead to lower levels of polyunsaturated PCs [35]. These findings suggest that reduced polyunsaturated PCs might result from lower PEMT activity during OSCC progression.

PE is the second most abundant phospholipids in mammalian membranes and contributes 20-30% to the total phospholipids content and is essential for the growth and stability of energy-producing organelles. Recent evidence indicates that plasmalogen phospholipids are particularly sensitive to oxidation and may possess antioxidative properties. It has been reported that plasmalogens had protective qualities against oxidative stress in cells [36]. The decreased pPE level in OSCC patients suggested that cancer cells might exhibit elevated oxidative stress. Two of pPEs, PE(P-36:4) and PE(P-38:5), had step-wise decrease with the development and progression, which was consistent with previous findings that oxidative stress was associated with cancer progression [37].

Five sphingolipids were increased in OSCC patients compared to normal controls. Bioactive sphingolipid metabolites served as an important lipid second messengers in the regulation of tumor cell growth, differentiation and angiogenesis [38], which were composed of hydrophilic head groups, such as sphingomyelin (SM), ceramide-1-phosphate and glucosylceramide (GlcCer) [39]. Ceramideis thought to be induce death, growth inhibition and senescence in cancer cells and can be synthesized through multiple different pathways within the cell. It could be catalyzed the conversion of sphingosine to sphingosine 1-phosphate (S1P) by sphingosine kinases (SPHKs). S1P is usually considered to promote the survival of cells [40, 41]. In recent years, many efforts have been made to elucidate the molecular signaling pathways by which ceramide and S1P cause their effect and these studies also reveal important roles for ceramide and S1P across cancers [42]. Ceramide regulation is increasingly implicated in cancer pathogenesis and prognosis. SM has been reported to participate in many cellular processes, such as promoting cell proliferation and differentiation. If SM is produced under physiological and pathological conditions, it can activate various signaling cascades. Guo et al. identified that SM associated with the lungs cancer progressions [43]. Sphingolipid metabolism plays a number of key roles in the response of cancer to therapy. Translating the scientific principles of sphingolipid metabolism in to realistic strategies would improve the cancer treatment outcomes.

In summary, we found that that the OSCC patients and control groups can be discriminated based on lipid profiling and the differential analysis of OSCC patients and controls is remarkable. Furthermore, the lipids associated with pathological staging were identified. The results may provide additional information on lipid metabolism in OSCC that allows us to more deeply understand oncogenesis. These findings also have potential clinical utility in the future if confirmed in larger studies.

## MATERIALS AND METHODS

### Hypothesis

We hypothesize that OSCC patients have distinct lipid profiles that reflect the disease progression and that these distinctive lipid profiles influence lipid homeostasis, which is reflected in the plasma. Therefore, determining the concentrations of specific lipids would reflect the existence and progression of OSCC. By comparing the plasma concentrations of lipids among populations with and without cancer or among those with different stages of OSCC, a few lipids that are most representative of the disease status would be identified as plasma lipid biomarkers in the diagnosis of OSCC.

### Sample collection

Patients were consecutively and prospectively included in the study when admitted for surgery for a clinically suspicious malignant OSCC at the Department of Oral and Maxillofacial Surgery, The Affiliated Second Hospital of Harbin Medical University, Harbin, Heilongjiang Province, China, from March 2013 to July 2015. The inclusion and exclusion criteria for participants and the sample collection procedures were described as follows: participants involved in this study were not taking any medications, and those suffering from metabolic diseases, liver diseases, kidney diseases or any other types of cancer were excluded. The blood samples for biomarker measurements were obtained by routine venipuncture prior to surgery. Samples were maintained at room temperature during transportation and then centrifuged within 30 minutes after collection. The isolated plasma samples were stored at -80°C for further analysis. This study was approved by the Ethics Committee of the second hospital of Harbin Medical University.

### Sample preparation

All the plasma samples were thawed at 4°C, and 30 μl of plasma was then mixed with 90 μl of precooled methanol. Next, 300 μl of methyl tert-butyl ether (MTBE) was added to the mixture, which was oscillated at 1000 rpm in 25°C for 1 hour, followed by the addition of 75 μl of deionized water. The samples were then mixed by vortexing for 1 min and oscillated at 1000 rpm for 10 min at 4°C, followed by centrifugation at 12000PM. A total of 240 μl from the upper layer was transferred into a clear vial and dried in a vacuum rotary dryer. The residue was dissolved in 100 μl of a 50/50 (v/v) solution of isopropanol/methanol for analysis. To ensure the stability and repeatability of the UPLC-MS, a total of 15 QC samples were prepared and used in this study. Pooled QC samples were prepared by mixing samples from 15 OSCC patients, 50 normal subjects. The preparation of the QC sample was the same as that was for the experimental samples.

### Chromatography

A 5 μl aliquot of the pre-treated sample was injected into a Kinetex Core-shell Silica C18 2.1 mm×50 mm, 1.3 μm column (Phenomenex, Torrance, CA, USA) on a UPLC system (Waters, Milford, USA). The mobile phase consisted of 10/90 (v/v) acetonitrile/isopropanol (solvent A) and 60/40 (v/v) acetonitrile/deionized water (solvent B). The flow rate was set at 0.26 ml/min with a column temperature of 40°C.

A linear mobile phase gradient was used as follows: 10% A, held for 1 min; 1.0–8.0 min, increased to 30% A; 8.0–18.0 min, increased to 75% A; 18.0–20.0 min, increased to 97% A; 20.0–24.0 min, maintained at 97% A; 24.0–25.0 min, decreased to 10% A; and 25.0-26.4 min, held at 10% A. After each analytical run, the mobile phase was returned to 1% A in 0.1 min and equilibrated at 1% A for 1 min. To minimize the analytical variation, all the samples were randomly analyzed in succession. In addition, QC samples were analyzed at the beginning and the end of each batch run to ensure stability during analysis.

### Mass spectrometry

Data acquisition was performed with an Agilent 6520-QTOF instrument (Agilent Technologies) equipped with an electrospray ionization source operating in ESI+ mode. The capillary voltage was 4.0 kV. Nitrogen was used as the dry gas, and the desolvation gas flow was set at 10 L/min. The desolvation temperature was set at 330°C. Centroid data were collected in the full scan mode from 50 to 1000 m/z.

### Data preprocessing and annotation

Raw data were converted into mzdata-format files using MassHunter Qualitative Analysis Software (Agilent Technologies), and these files were then imported into the XCMS package in the R platform for preprocessing. The following parameters were set as default values in the XCMS function: xcmsSet (method=“centWave”, peakwidth5c (5, 20)); group (bw5); rector (method5“obiwarp”). The preprocessing results generated a data matrix that consisted of the retention time (RT), mass-to-charge ratio (m/z) values, and peak intensity. The CAMERA package in R was used to annotate isotope peaks, adducts and fragments in the peak lists. Isotopic peaks were excluded prior to statistical analysis.

### Statistical analysis

The continuous variables in patients with OSCC and the controls were presented as the median and range, whereas the categorical variables were presented as a frequency for each category. PCA was performed to determine the detection of stability and the replication. To visualize the discrimination performance of the lipid profiling between OSCC and controls, we performed PLS-DA with mean centering and unit variance scaling of each variable. The parameters of the model, such as R^2^Y and Q^2^, were analyzed to avoid the risk of over-fitting. The differences in the concentrations of lipids in patients with OSCC and controls were compared using the Wilcoxon rank-sum test. To account for the multiple test issue, lfdr, which was calculated with the R package “fdrtool”. Multivariate VIP values were calculated for each variable. The potential biomarkers were selected based on the criteria of lfdr<0.05 and VIP>1. To facilitate the clinical utility of these potential biomarkers for future clinical practice, we utilized Student's t-test to select the staging related lipids used for early diagnosis. The intensity of lipids associated with pathological staging is presented using a box plot. Statistical analysis was performed in the R platform.

## SUPPLEMENTARY MATERIALS FIGURES



## Abbreviations

OSCC: oral squamous cell carcinoma
UPLC-MS: ultra-performance liquid chromatography mass spectrometry
LN: lymph node
SCCA: serum squamous cell carcinoma antigen
PC: phosphatidylcholines
GPs: glycerphospholipids
PEs: glycerophosphoethanolamines
PG: glycerophosphoglycerols
Lyso-PC: lyso-glycerophosphocholines
SPs: sphingolipids
Cers: ceramides
SMs: sphingomyelins
pPEs: PEs with plasmalogen
LPA: lysophosphatidic acid
PEMT: phosphatidylethanolamine *N*-methyltransferase
SM: sphingomyelin
GlcCer: ceramide-1-phosphate and glucosylceramide
S1P: sphingosine 1-phosphate
SPHKs: sphingosine kinases
QC: quality control
ESI: electrospray ionization
ROC: receiver operating characteristic
m/z: measured mass to charge ratio
RT: retention time
