# Supplementary material for: Plasma lipid profiling and diagnostic biomarkers for oral squamous cell carcinoma
Source: Oncotarget. 2017 Sep 27;8(54):92324–32. doi: 10.18632/oncotarget.21289 (PMC5696184; doi:10.18632/oncotarget.21289)
Supplement: Supplementary file 1 [file oncotarget-08-92324-s001.pdf]

Plasma lipid profiling and diagnostic biomarkers for oral squamous cell carcinoma

SUPPLEMENTARY MATERIALS

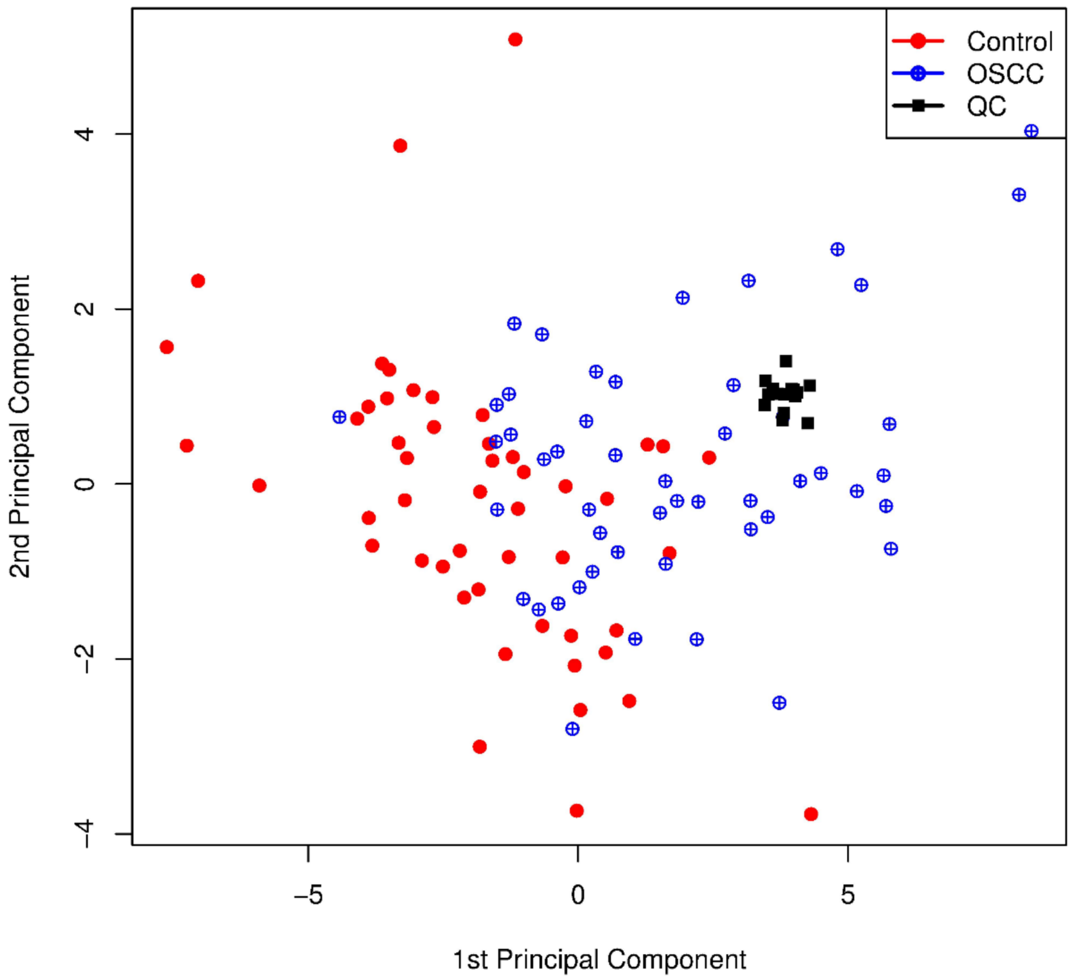

Supplementary Figure 1: PCA score plot for discriminating OSCC and control.

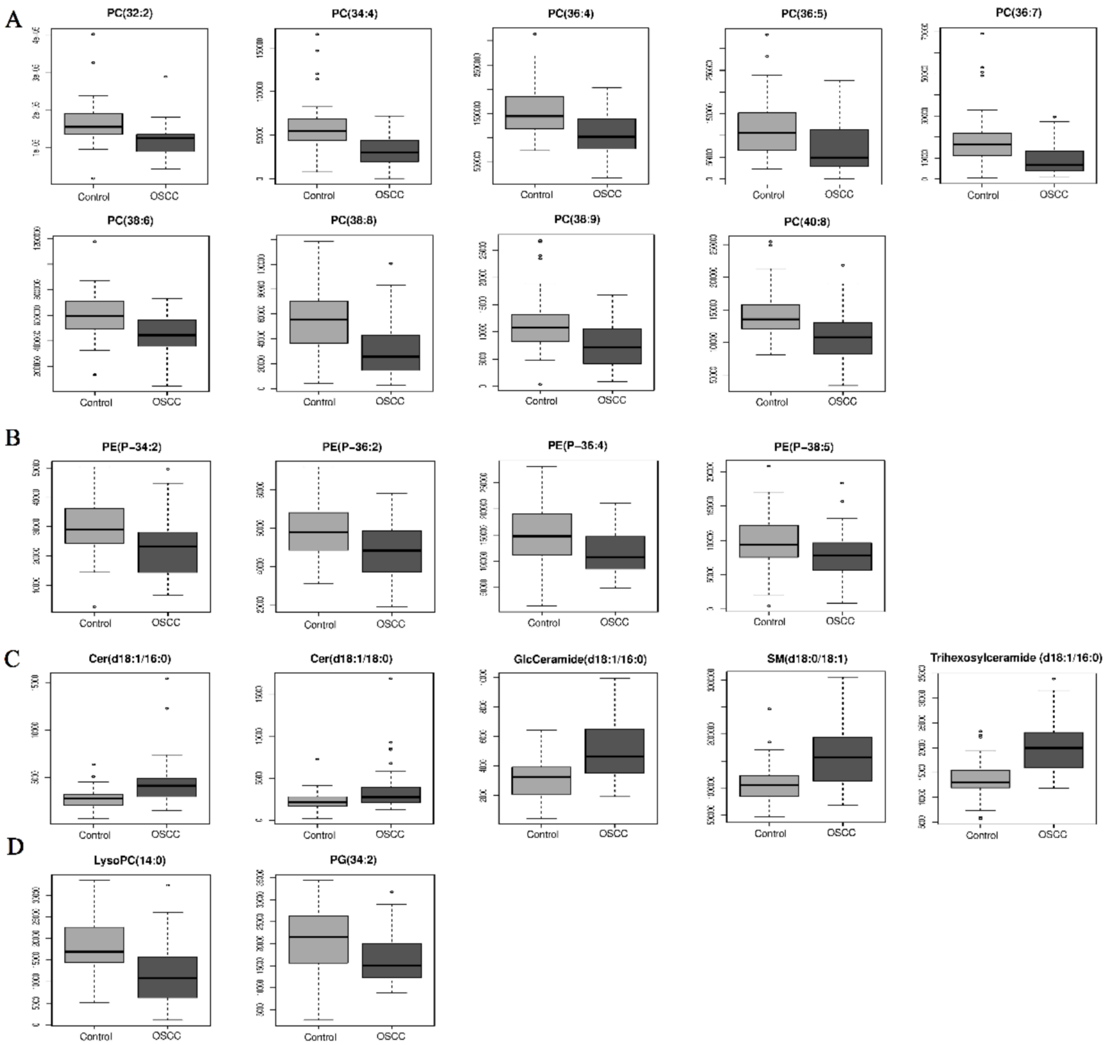

Supplementary Figure 2: The intensity levels of lipids associated with OSCC and control. **(A)** phosphatidylcholines; **(B)** glycerophosphoethanolamines; **(C)** sphingolipids; **(D)** lyso-glycerophocholines and glycerophosphoglycerols.

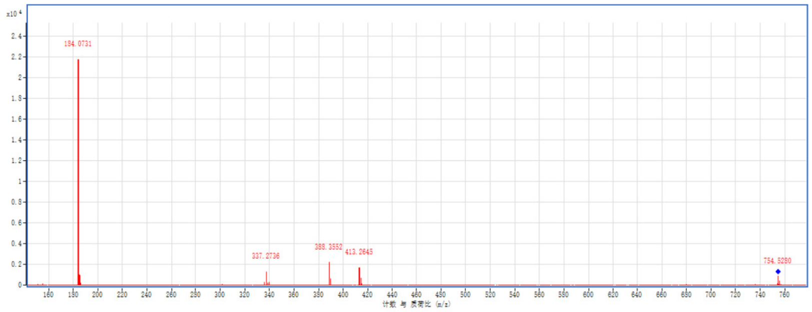

Supplementary Figure 3: The MS/MS spectrum of m/z 754.5129 (PC(34:4)) at 13.56 min in a plasma sample.

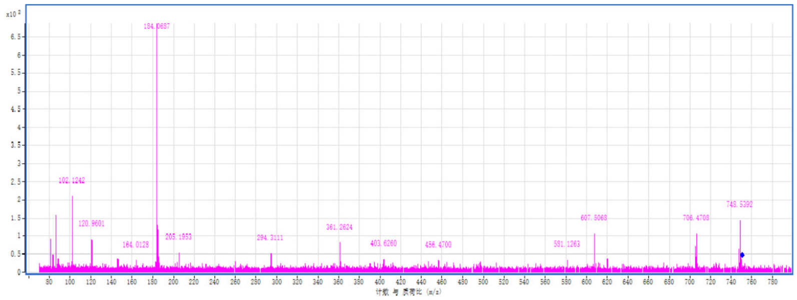

Supplementary Figure 4: The MS/MS spectrum of m/z 750.4511 (PE(P-38:5)) at 15.57min in a plasma sample.

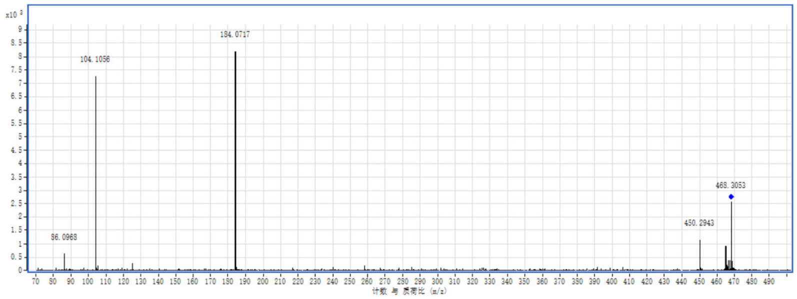

Supplementary Figure 5: The MS/MS spectrum of m/z 468.3542 (LysoPC(14:0)) at 1.86 min in a plasma sample.

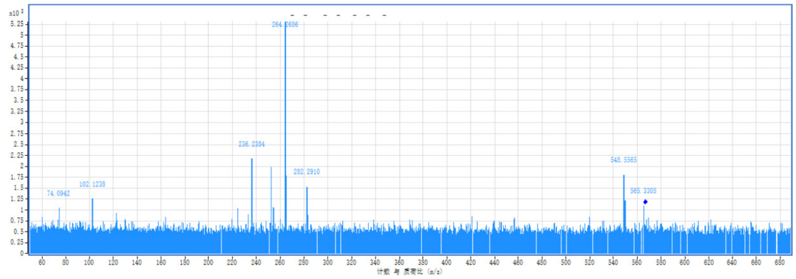

Supplementary Figure 6: The MS/MS spectrum of m/z 566.5151 (Cer(d18:1/18:0)) at 16.79min in a plasma sample.

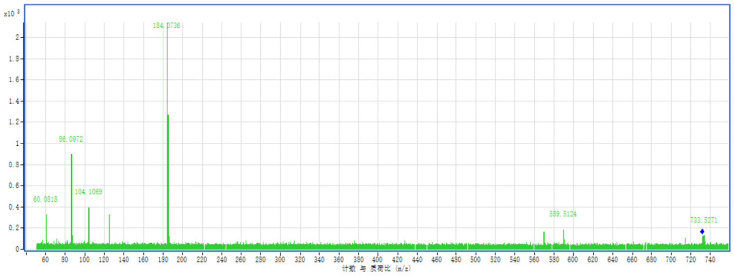

Supplementary Figure 7: The MS/MS spectrum of m/z 731.6491 (SM(d18:0/18:1)) at 15.42 min in a plasma sample.
